# Ethanolic Extract of Taheebo Attenuates Increase in Body Weight and Fatty Liver in Mice Fed a High-Fat Diet

**DOI:** 10.3390/molecules191016013

**Published:** 2014-10-08

**Authors:** Won Hee Choi, Min Young Um, Jiyun Ahn, Chang Hwa Jung, Myung Kyu Park, Tae Youl Ha

**Affiliations:** 1 Research Group of Nutrition and Metabolic System, Korea Food Research Institute, Seongnam 463-746, Korea; E-Mails: kfri2@hanmail.net (W.H.C.); minyoungum@gmail.com (M.Y.U.); jyan@kfri.re.kr (J.A.); chjung@kfri.re.kr (C.H.J.); 2 Division of Food Biotechnology, University of Science and Technology, Daejeon 305-350, Korea; 3 Erom Life Science Corporation Corporation/R&D Center, Seongnam 443-702, Korea; E-Mail: baduk60@erom.co.kr

**Keywords:** Taheebo, *Tabebuia avellanedae*, obesity, lipid metabolism

## Abstract

We evaluated whether intake of an ethanolic extract of Taheebo (TBE) from *Tabebuia avellanedae* protects against body weight increase and fat accumulation in mice with high-fat diet (HFD)-induced obesity. Four-week old male C57BL/6 mice were fed a HFD (25% fat, w/w) for 11 weeks. The diet of control (HFD) mice was supplemented with vehicle (0.5% sodium carboxymethyl cellulose by gavage); the diet of experimental (TBE) mice was supplemented with TBE (150 mg/kg body weight/day by gavage). Mice administered TBE had significantly reduced body weight gain, fat accumulation in the liver, and fat pad weight, compared to HFD mice. Reduced hypertrophy of fat cells was also observed in TBE mice. Mice administered TBE also showed significantly lower serum levels of triglycerides, insulin, and leptin. Lipid profiles and levels of mRNAs and proteins related to lipid metabolism were determined in liver and white adipose tissue of the mice. Expression of mRNA and proteins related to lipogenesis were decreased in TBE-administered mice compared to mice fed HFD alone. These results suggest that TBE inhibits obesity and fat accumulation by regulation of gene expression related to lipid metabolism in HFD-induced obesity in mice.

## 1. Introduction

Obesity is defined as abnormal or extensive fat accumulation that negatively affects health [1] and is associated with numerous chronic diseases, including type 2 diabetes, hypertension, cardiovascular disease, and nonalcoholic fatty liver disease (NAFLD), as well as psychological and social problems [2,3,4]. Obesity results from chronic caloric intake in excess of energy expenditure, a common occurrence in the modern environment that facilitates consumption of hypercaloric diets, including high-fat diet (HFD) [5], and which ultimately accumulates fat, primarily in the liver. In particular, excessive fat accumulation in the liver caused by obesity in general dysregulates insulin action in the liver, leading to insulin resistance [6]. Recent prospective diet intervention studies indicate that 5%–10% weight loss improves liver histology and reduces hepatic triglycerides [7,8].

Through the process of adipogenesis, preadipocytes are converted to adipocytes, and fat accumulation is induced in the tissues. Peroxisome proliferator-activated receptor γ2 (PPARγ2) and CCAAT/enhancer binding protein α (C/EBPα) are the master transcriptional regulators of the adipogenic process [9]. Acetyl-CoA carboxylase (ACC) and fatty acid synthase (FAS) are known to be regulated by sterol regulatory element-binding protein-1c (SREBP-1c), which is a critical transcription factor that stimulates lipogenic enzymes involved in lipid synthesis [10]. Hepatic lipid accumulation is caused by the upregulation of *de novo* lipid synthesis, activation of lipid uptake, and suppression of lipid catabolism. Also, increased *de novo* lipogenesis in the liver accumulates excessive triglyceride in the liver [10]. Activation of adipocyte lipid binding protein (aP2) and FAS by SREBP-1c leads to lipid accumulation in the tissues. Thus, inactivation of these adipogenic regulators or inhibition of expression of lipogenic genes may contribute to suppress adipogenesis and lipogenesis, and ultimately prevent obesity and fatty liver. Many studies have been conducted in an effort to identify novel anti-obesity agents that have the ability to control adipogenesis and lipogenesis.

Taheebo (TBE), obtained from the purple inner bark of the Bignoniaceae tree *Tabebuia avellanedae* Lorentz ex Griseb, which is found in tropical rain forests in northeastern Brazil, has been used as a traditional medicine for various diseases for more than 1500 years [11]. Recently, various fractions and extracts of *T. avellanedae* bark have been prepared and reported to have anti-inflammatory, antibacterial, and antifungal, as well as anticancer effects [12,13,14]. Although the pharmacological activity of the *T. avellanedae* bark has been investigated worldwide, its anti-obesity properties have not been studied to date. Therefore, in this study, we investigated the effect of an ethanolic extract of TBE on obesity and fatty liver in the HFD-induced obese mouse model.

## 2. Results and Discussion

### 2.1. Effect of TBE on Body Weight and Fat Tissue Weight and Serum Biochemical Parameters

**Figure 1:**
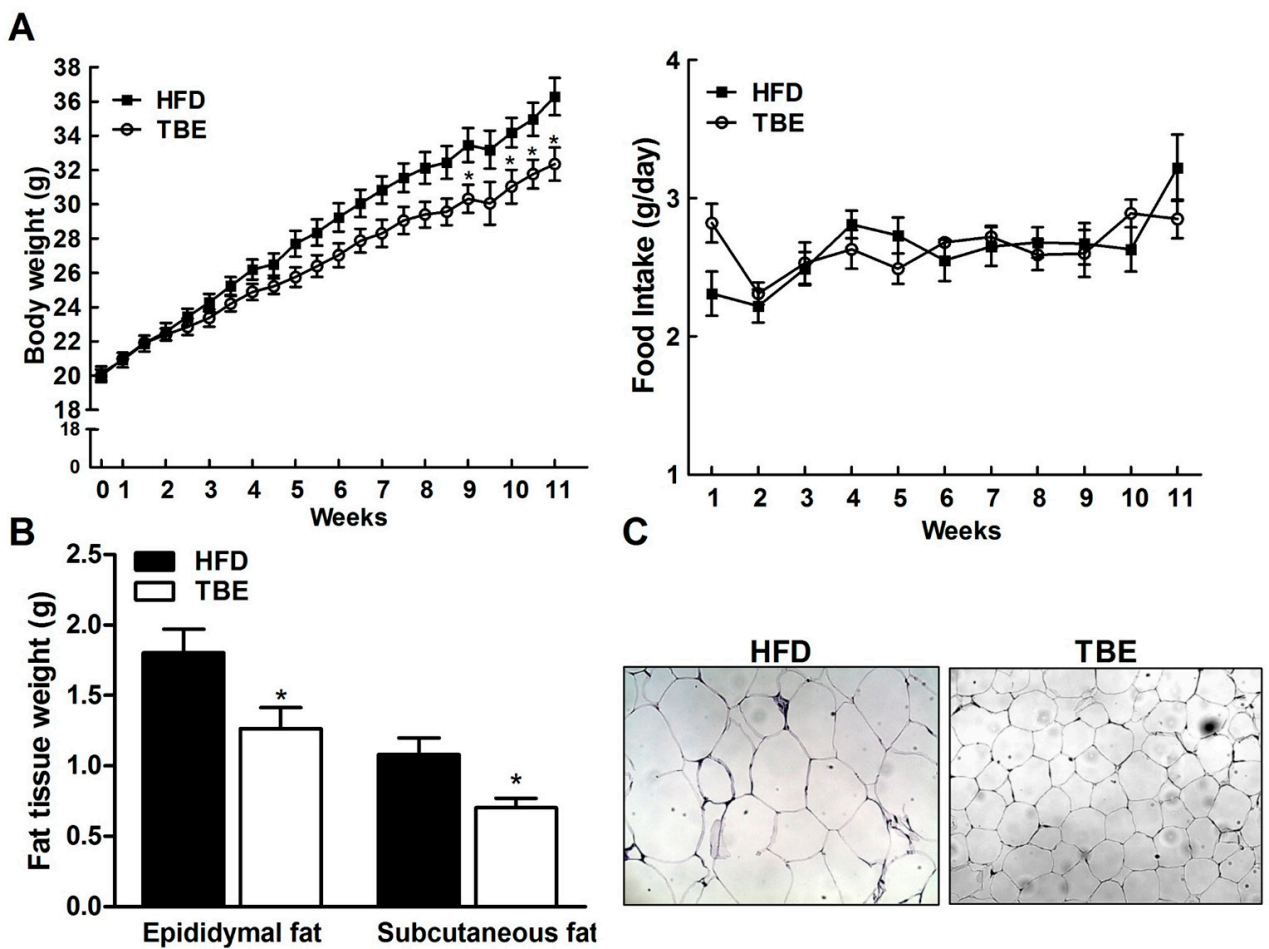
Effect of TBE on body weight and fat tissue weight. (**A**) Body weight (**left**) and food intake (**right**) during study period. (**B**) Fat tissue weight in the experimental mice. (**C**) Representative images of hematoxylin and eosin (H&E) staining of sections of epididymal fat tissue (original magnification, ×200). Data are mean ± standard error of the mean (SEM) (n = 10 per group). * *p <* 0.05 compared to the HFD group.

Figure 1A shows the effect of TBE on body weight of the mice fed an HFD for 11 weeks. Although there was no significant difference in food intake, a significant decrease in mean body weight was observed in the TBE group compared to the HFD group after 9 weeks of feeding. After 11 weeks of feeding the experimental diet, final mean body weight of mice in the TBE group was significantly lower than in the HFD group (36.29 ± 1.28 g *vs.* 32.35 ± 0.97 g) and body weight gain in the TBE-fed group was less than in the HFD-fed group by 19.98%. Moreover, the weights of epididymal and subcutaneous fat in the TBE group were significantly decreased compared to those of the HFD group (Figure 1B, *p <* 0.05), and hematoxylin and eosin (H&E) staining of epididymal fat tissue revealed that TBE supplementation reduced the size of adipocytes in HFD-fed mice (Figure 1C, *p <* 0.05), indicating that the reduction in body weight associated with TBE consumption might be the result of reduced adipose tissue weight.

We also analyzed serum markers of lipid metabolism. Overall, indicator levels were ameliorated by TBE consumption and, in particular, the levels of triglyceride, insulin, and leptin were significantly decreased compared to those of the HFD group (Table 1, *p <* 0.05).

**Table 1:** Effect of TBE on serum biochemical markers in HFD-fed mice.

Marker	HFD	TBE
Triglyceride (mg/mL)	68.96 ± 5.14	55.11 ± 1.69 *
Total cholesterol (mg/mL)	146.8 ± 7.92	134.4 ± 5.27
HDL-cholesterol (mg/mL)	63.12 ± 10.00	66.46 ± 6.69
Insulin (ng/mL)	1.73 ± 0.28	0.83 ± 0.15 *
Leptin (ng/mL)	15880 ± 2803	6829 ± 1420 *

Data are mean ± SEM (n = 10 per group). * *p <* 0.05 compared to the HFD group.

### 2.2. Effect of TBE on the Expression of Adipogenic Genes in the White Adipose Tissue (WAT)

**Figure 2:**
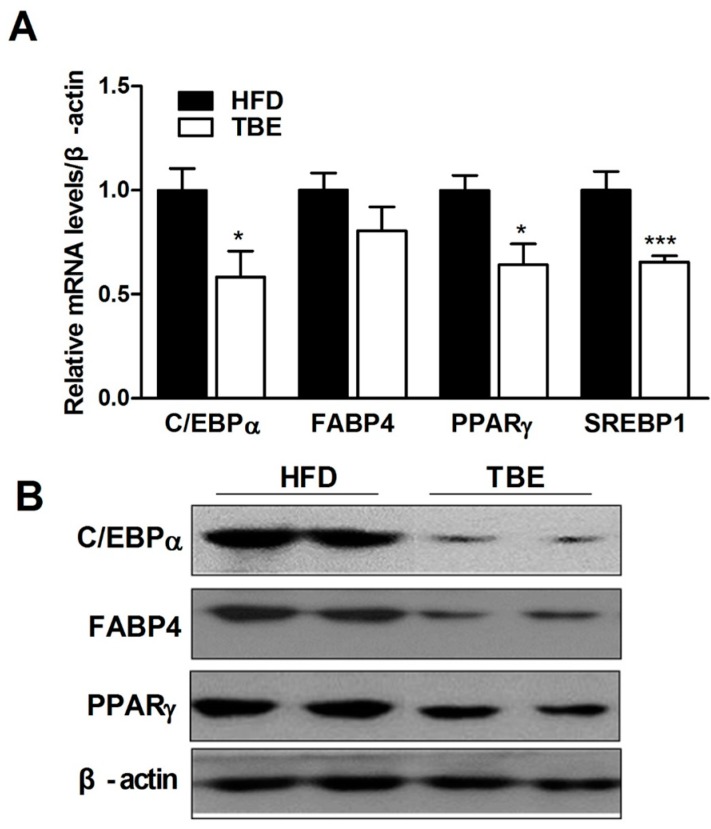
Effect of TBE on the expression of adipogenic genes in WAT. (**A**) Quantitative real-time PCR (qRT-PCR) analysis of the indicated adipogenic mRNAs. Relative mRNA levels were calculated after normalization of values to that of β-actin and presented as a ratio compared with HFD. (**B**) Western blot analysis of the indicated adipogenic proteins. Data are mean ± SEM (n = 10 per group). * *p* < 0.05, *** *p* < 0.001 compared to the HFD group.

Consistent with the reduced fat tissue weight and adipocyte size with TBE supplementation, the expression of mRNA and protein related to adipogenesis was suppressed by TBE supplementation. 3+6C/EBPα, FABP4, PPARγ and SREBP1c mRNA were each decreased to 1.7-, 1.25-, 1.56-, and 1.53-fold levels. Adipogenic proteins including PPARγ, C/EBPα, and fatty acid binding protein 4 (FABP4), were also suppressed by TBE supplementation, indicating that the anti-obesity effect of TBE might be the result of inhibition of adipogenesis (Figure 2A,B).

### 2.3. Effect of TBE on Lipid Content and Expression of Lipogenic Genes in the Live

**Figure 3:**
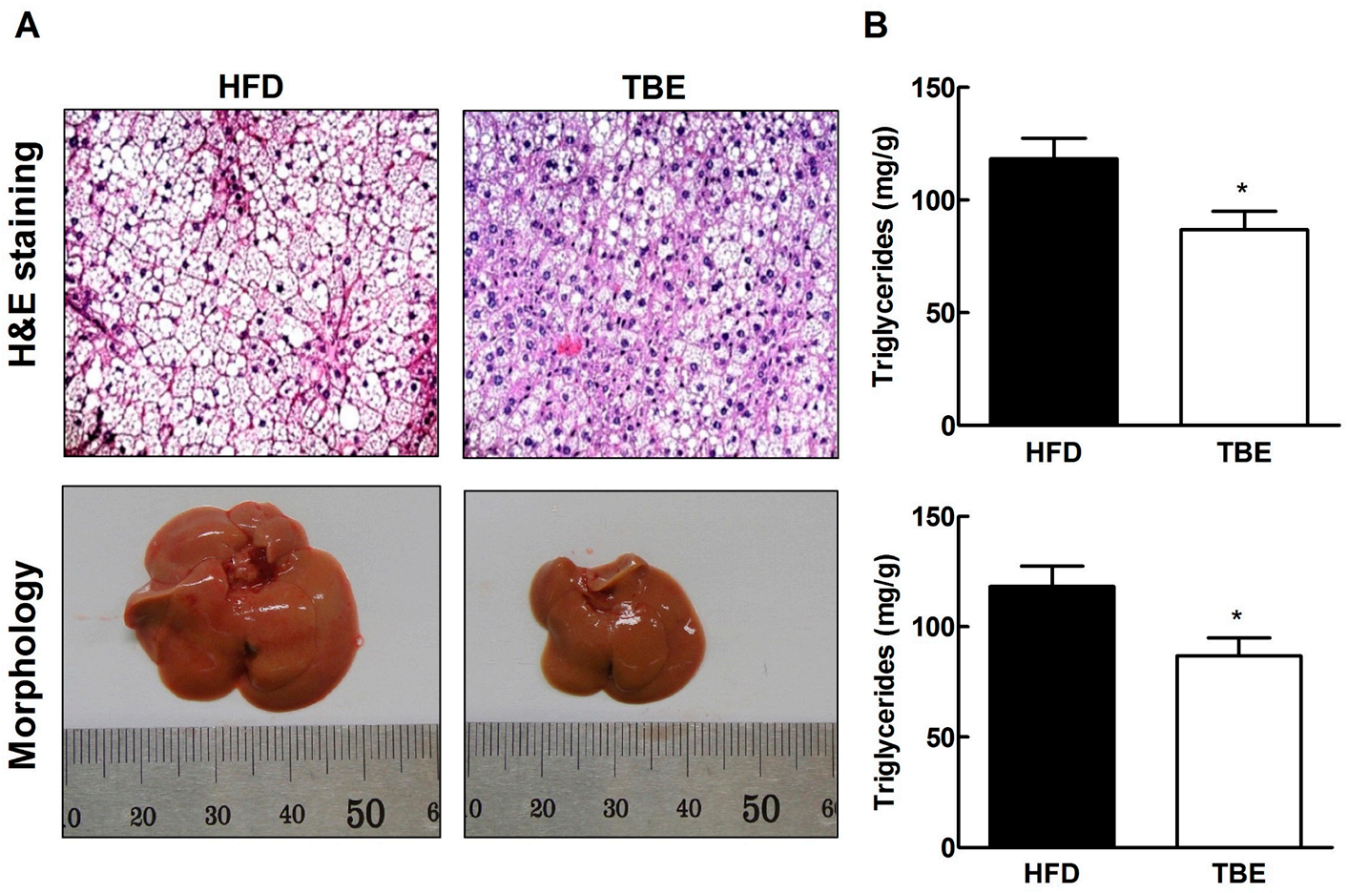
Effect of TBE on hepatic lipid accumulation. (**A**) Representative images of H&E-stained sections and gross morphology of livers (original magnification, ×200). (**B**) Total lipid and triglyceride contents of livers of HFD-fed mice. Data are mean ± SEM (n = 10 per group). * *p* < 0.05 compared to the HFD group.

**Figure 4:**
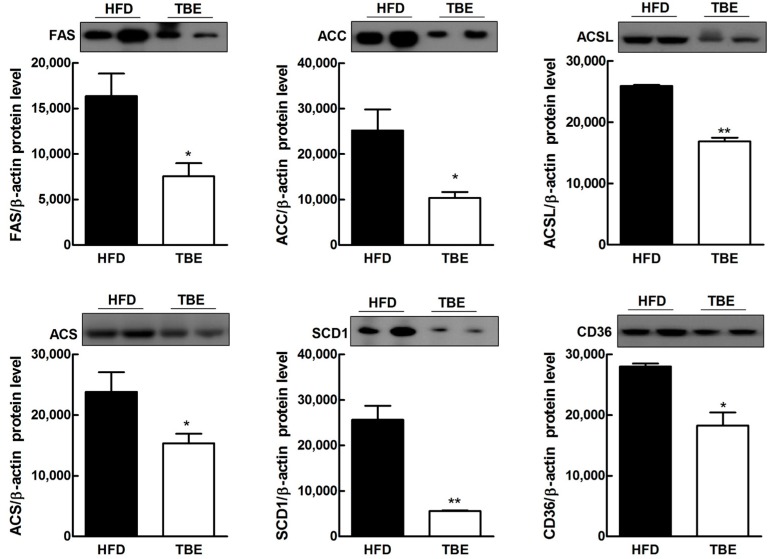
Effect of TBE on expression of proteins related to lipid metabolism by western blot analysis in livers of HFD-fed mice. Data are mean ± SEM (n = 10 per group*).* * *p* < 0.05, ** *p* < 0.01 compared to HFD group.

To examine whether TBE affected hepatic lipid accumulation, we investigated the histopathological changes in the liver. Histopathological analyses following H&E staining, and morphological analysis revealed smaller lipid droplets in the liver sections and reduced liver size of TBE group compared to the HFD group, suggesting that TBE supplementation effectively attenuated hepatic lipid accumulation (Figure 3A). These findings are consistent with lipid in the livers. Hepatic total lipid and triglyceride contents were significantly lower in the TBE group than in the HFD group (Figure 3B). To further explore the effect of TBE on hepatic lipid accumulation, we evaluated expression of genes related to lipid metabolism (Table 2 and Figure 4). Comparison of hepatic mRNA in the HFD and TBE groups indicated that TBE supplementation significantly reduced lipogenic mRNAs, such as FAS, acetyl CoA-synthetase (ACS), and stearoyl-CoA desaturase-1 (SCD1), providing further evidence of the antilipogenic effect of TBE. Consistent with reduced expression of lipogenic mRNAs, the expression of lipogenic proteins, such as FAS, ACS, SCD1, ACSL, and cluster of differentiation 36 (CD36) were also downregulated in the TBE group compared to the HFD group. These results indicate that TBE supplementation attenuates lipid accumulation in the liver through the regulation of lipogenesis.

**Table 2:** Effect of TBE on expression of mRNAs related to lipid metabolism in the livers of the HFD-fed mice.

Genes	Relative mRNA Levels	
	HFD	TBE
ACSL	1.00 ± 0.123	0.8237 ± 0.084
ACS	1.00 ± 0.036	0.7346 ± 0.015 **
GPAM	1.00 ± 0.723	0.9089 ± 0.101
FAS	1.00 ± 0.063	0.7141 ± 0.044 *
SCD1	1.00 ± 0.057	0.6109 ± 0.0742 **

Data are mean ± SE (n = 10 per group). * *p* < 0.05, ** *p* < 0.01 compared to the HFD group. Relative mRNA levels were calculated after normalization of values to that of β-actin and presented as a ratio compared with HFD.

### 2.4. Discussion

TBE obtained from *T. avellanedae* is used as a traditional medicinal for various diseases. Previous studies have reported that TBE exerts various pharmacological actions, including anti-inflammatory, antibacterial, and antifungal, as well as anticancer effects [12,13,14]. However, no study has investigated the effect of TBE in diet-induced obesity. Therefore, we examined whether TBE extract has an anti-obesity effect and potential as a natural anti-obesity supplement.

In the present study, we evaluated the anti-obesity effect of TBE in HFD-induced obese mice. During 11 weeks of feeding a HFD, a smaller increase in body weight was observed in the TBE group than in the HFD group. In particular, epididymal and subcutaneous fat mass was reduced significantly by TBE consumption. Consistent with these results, C/EBPα and PPARγ mRNA and protein levels were also downregulated in the WAT in the TBE group. In the process of adipogenesis, C/EBPα and PPARγ are key transcriptional factors and mediate the transcription of terminal adipocyte differentiation marker genes [15]. Taken together, our findings suggest that TBE attenuates HFD-induced increase in body weight by inhibition of adipogenesis.

It has been suggested that leptin contributes to hepatic steatosis by increasing fatty acid concentration in the liver and promoting insulin resistance [16]. In this study, TBE supplementation was found to significantly reduce serum leptin and insulin levels in mice fed a HFD. Moreover, TBE supplementation also markedly lowered hepatic total lipid and triglyceride levels in mice fed a HFD. This finding was supported by histologic analysis of liver tissue, and indicates that TBE supplementation reduced lipid accumulation in the liver. To understand the underlying molecular mechanisms, we assessed the expression of hepatic genes regulating lipid metabolism. The liver is a major site of lipogenesis, where most lipogenic genes, including FAS and SCD1, are highly expressed [17]. Moreover, numerous studies have reported that hepatic expression of FAS and SCD1 was increased in obese mice [18]. FAS, which is a central lipogenic gene, provides nonesterified fatty acid substrate for triglyceride synthesis and increases fatty acid uptake with CD36 [19]. CD36, a target gene of PPARγ, may promote fatty liver by accelerating fatty acid transport to the liver [20]. Suppressed fatty acid uptake into the liver, which was mediated in part by decreased expression of CD36, may help suppress fat deposition in the liver. SCD1 is a regulatory enzyme in lipogenesis, catalyzing the rate-limiting step in the overall *de novo* synthesis of monounsaturated fatty acid (MUFA), mainly oleate and palmitoleate from stearoyl- and palmitoyl-CoA [17]. MUFAs synthesized by SCD1 are the major substrates for the synthesis of various lipids, such as phospholipids, triglycerides, and cholesterol esters [21]. In our study, TBE supplementation markedly suppressed lipogenic gene (FAS, ACC, ACSL, ACS, SCD1 and CD36) expression. Taken together, our findings suggest that TBE reduces fatty liver by regulating genes related to lipid metabolism.

According to the recent literature, flavonoids, cyclopentene dialdehydes, benzoic acid, quinones, furanonaphthoquinones, and naphthoquinones have been identified as a major compounds in *T. avellanedae* [11,12,22]. Among the naphthoquinones, β-lapachone appears to have clinical importance [11,12], and Hwang reported that β-lapachone ameliorates metabolic abnormalities [23]. Whereas we explained several mechanisms underlying the anti-obesity effect of TBE, further investigation of the active compounds responsible for this effect is needed. Collectively, our study provides evidence that TBE supplementation exerts an anti-obesity effect and inhibits fatty liver by regulating expression of genes related to lipid metabolism.

## 3. Experimental Section 

### 3.1. Preparation of TBE

To prepare TBE, dried inner bark of *T. avellanedae* was extracted three times with 70% ethanol at room temperature [11]. Extracts were pooled, filtered, and concentrated under reduced pressure at 40 °C, with a final yield of 12%. The extract was suspended in 0.5% sodium carboxymethyl cellulose (CMC-Na) immediately prior to the start of the experiments.

### 3.2. Animal Models

Four-week old male C57BL/6 mice were purchased from Orient Bio Inc. (Gyeonggi, Korea). The animals were maintained at a temperature and humidity of 21–25 °C and 50%–60%, respectively, and kept on a 12-h light/12-h dark cycle with free access to food and water. After one week of adaptation, all mice were fed a HFD containing (by weight) 20% casein as protein, 25% fat, and 44.5% carbohydrate supplemented with 0.5% cholesterol *ad libitum* for 11 weeks. Control (HFD) mice were orally administered vehicle (0.5% CMC-Na in distilled water) and experimental (TBE) mice were orally administered TBE extract (150 mg TBE/kg body weight). HFD were based on the American institute of nutrition-76 (AIN-76) diet formula. All procedures involving animals were conducted in accordance with the Guidelines for Institutional Animal Care and Use Committee of the Korea Food Research Institute (KFRI-IACUC, KFRI-M-13021).

### 3.3. Tissue Analysis

At the end of the study period, mice were fasted for 12 h and sacrificed under anesthesia. Blood was collected from the abdominal aorta and centrifuged at 1500× *g* for 15 min to separate the serum. Triglycerides, total cholesterol, and HDL cholesterol levels in serum were measured using commercial enzyme kits (Shinyang Chemical Co., Seoul, Korea). Liver tissue and fat tissues were also excised and weighed. Hepatic lipids were extracted according to the method described by Folch *et al.* (1957). Hepatic triglyceride content was measured using commercial enzyme kits (Shinyang Chemical Co.).

### 3.4. Histopathological Evaluation

Liver and epididymal fat tissues were fixed in 10% neutral-buffered formalin, embedded in paraffin, and 5-μm sections were prepared. Liver and epididymal fat sections were stained with H&E. Pathological changes were investigated using an Olympus (Tokyo, Japan) BX50 light microscope to confirm lipid droplet and adipocyte size.

### 3.5. qRT-PCR Analysis

Total RNA from the livers and epididymal fat tissues were extracted using NucleoSpin RNA II (Macherey-Nagel, Düren, Germany) according to the manufacturer’s instructions, and reverse transcription to generate cDNA was performed using the iScript^TM^ cDNA Synthesis Kit (BioRad, Hercules, CA, USA) according to the manufacturer’s instructions. qPCR was performed with 1 μg of cDNA, 10 μL of SYBR Green Master Mix (Toyobo, Tokyo, Japan) and forward/reverse primers in a StepOnePlus Real-Time PCR System (Applied Biosystems, Foster City, CA, USA). The cycling conditions were as follows: 50 °C for 2 min and 95 °C for 5 min, followed by 40 cycles of 95 °C for 5 s, 55–60 °C for 10 s, and 72 °C for 15 s. The melting curve was also analyzed to ensure that only a single product was amplified and the condition were as follows: 95 °C for 15 s, 60 °C for 1 min, and 95 °C for 15 s. The sequences of the primers used in this study are shown in Table 3. Relative mRNA expression levels were calculated after normalization of values to that of β-actin.

**Table 3:** Primer sequences for qRT-PCR.

Primer	Direction	Sequence
FAS	Forward Reverse	5'-GGAGGTGGTGATAGCCGGTAT-3' 5'-TGGGTAATCCATAGAGCCCAG-3'
SCD1	Forward Reverse	5'-TTCTTGCGATACACTCTGGTGC-3' 5'-CGGGATTGAATGTTCTTGTCGT-3'
ACSL	Forward Reverse	5'-CTCACCATTATATTGCTGCCTGT-3' 5'-TCTCTTTGCCATAGCGTTTTTCT-3'
ACS	Forward Reverse	5'-AAACACGCTCAGTAGCACCAC-3' 5'-AGCCAAGTAGGAAGCTCTCTC-3'
GPAM	Forward Reverse	5'-ACAGTTGGCACAATAGACGTTT-3' 5'-CCTTCCATTTCAGTGTTGCAGA-3'
PPARγ	Forward Reverse	5'-TCGCTGATGCACTGCCTATG-3' 5'-GAGAGGTCCACAGAGCTGATT-3'
C/EBPα	Forward Reverse	5'-CAAGAACAGCAACGAGTACCG-3' 5'-GTCACTGGTCAACTCCAGCAC-3'
FABP4	Forward Reverse	5'-AAGGTGAAGAGCATCATAACCCT-3' 5'-TCACGCCTTTCATAACACATTCC-3'
β-actin	Forward Reverse	5'-GCAGGAGTACGATGAGTCCG-3' 5'-ACGCAGCTCAGTAACAGTCC-3'

### 3.6. Western Blotting

Proteins were extracted from liver and epididymal fat tissues and quantified, followed by separation by 10% SDS-PAGE and transfer to polyvinylidene fluoride membranes (Millipore, Billerica, MA, USA). The membranes were blocked in 5% skim milk in Tris-buffered saline containing 0.05% Tween-20 (TBST) for 2 h at room temperature. After overnight incubation at 4 °C with primary antibodies, membranes were incubated with appropriate horseradish peroxidase-conjugated secondary antibodies for 1 h at room temperature. Immunodetection was carried out with Amersham ECL detection reagent (GE Healthcare, Chalfont St. Giles, UK). Antibodies used in this study were purchased from Santa Cruz Biotechnology (Santa Cruz, CA, USA) and Cell Signaling Technology (Danvers, MA, USA).

### 3.7. Statistical Analysis

The data were expressed as mean ± standard error of the mean. Differences between groups were examined for statistical significance using Student’s *t*-test and the 2-way ANOVA test was used to compare the weight changes between the groups using GraphPad Prism version 5.0 (GraphPad Software, San Diego, CA, USA), with a value of *p* < 0.05 selected as the threshold for statistical significance.

## 4. Conclusions 

In conclusion, an ethanolic extract of TBE from the purple inner bark of the Bignoniaceae tree *T. avellanedae* Lorentz ex Griseb attenuates obesity and fatty liver in HFD-induced obese mice by regulating expression of genes related to lipid metabolism.
